# The Role of Wild Boar as Host of Japanese Encephalitis Virus in the Absence of Domestic Pigs

**DOI:** 10.3390/v16081273

**Published:** 2024-08-09

**Authors:** Fuka Kikuchi, Ai Hayashi, Karen Yamada, Yusuke Matsui, Reiko Shimbashi, Yuji Noguchi, Kazunori Tachibana, Tetsuya Mizutani, Akihiko Tokaji, Akira Yoshikawa, Motoki Ihara, Kazunori Oishi, Hajime Kamiya, Satoru Arai, Motoi Suzuki

**Affiliations:** 1 Center for Infectious Diseases Epidemiology and Prevention Research, Tokyo University of Agriculture and Technology, Tokyo 183-8509, Japan; fkikuchi@niid.go.jp (F.K.); tmizutan@cc.tuat.ac.jp (T.M.); 2 Center for Surveillance, Immunization and Epidemiologic Research, National Institute of Infectious Diseases, Tokyo 162-8640, Japan; ahayashi@niid.go.jp (A.H.); kyamada@niid.go.jp (K.Y.); shimbashir@niid.go.jp (R.S.); toyamaeiken1@chic.ocn.ne.jp (K.O.); hakamiya@niid.go.jp (H.K.); mosuzuki@niid.go.jp (M.S.); 3 Tohoku University Graduate School of Medicine, Sendai 980-8575, Japan; 4 Department of Chemistry, Faculty of Science, Tokyo University of Science, Tokyo 162-8601, Japan; 5 Nagasaki Prefecture Tsushima Hospital, Nagasaki 817-0322, Japan; 6 Nagasaki Prefecture Kamitsushima Hospital, Nagasaki 817-1701, Japan; 7 Department of Health Policy, Kochi Public Health and Environmental Science Research Institute, Kochi 780-0850, Japan; akihiko_tokaji2@ken4.pref.kochi.lg.jp; 8 Department of Public Health, Nagasaki Prefectural Institute for Environmental Research and Public Health, Nagasaki 856-0026, Japan; a-yoshikawa@pref.nagasaki.lg.jp (A.Y.); mihara@pref.nagasaki.lg.jp (M.I.); 9 Toyama Institute of Health, 17-1 Nakataikouyama, Imizu, Toyama 939-0363, Japan

**Keywords:** Japanese encephalitis, seroprevalence, serosurvey, outbreak, boars

## Abstract

Pigs are the most common amplifying hosts of the Japanese encephalitis virus (JEV). In 2016, four residents on Tsushima Island who did not own pig farms were diagnosed with JE. Therefore, a serosurvey was conducted to estimate the risk and seroprevalence of JEV after the outbreak. Sera collected from 560 Tsushima Island residents between January and September 2017 were tested for neutralizing antibodies against JEV strains JaGAr01 (genotype 3) and Muar (genotype 5). Sera collected from six wild boars between June and July 2022 were tested. The seroprevalence rates of neutralizing antibodies against JaGAr01 and Muar were 38.8% and 24.6%, respectively. High anti-JEV neutralizing antibody titers of ≥320 were identified in 16 residents, including 3 younger than 6 years with prior JEV vaccination, 2 in their 40s, and 11 older than 70. However, no anti-JEV-specific IgM was detected. Residents who engaged in outdoor activities had higher anti-JEV antibody titers. Sera from wild boars were negative for JEV RNA, but four of six samples contained neutralizing antibodies against JEV. Therefore, JEV transmission continues on Tsushima Island, even in the absence of pig farms, and wild boars might serve as the amplifying hosts.

## 1. Introduction

Japanese encephalitis virus (JEV) belongs to the genus Orthoflavivirus of the family Flaviviridae, which includes three other genera (Hepacivirus, Pegivirus, and Pestivirus). All members of this virus family possess a single-stranded, positive-sense 9.2–11.0 kb RNA genome containing 5′—and 3′—untranslated regions (UTRs) between a single open reading frame with three structural proteins [capsid (C), precursor membrane (prM), and envelope (E)] and seven nonstructural proteins (NS1, NS2A, NS2B, NS3, NS4A, NS4B, and NS5) [1,2]. The incubation period of Japanese encephalitis (JE) is 5 to 15 days, during which the virus may cause various clinical symptoms [3,4,5]. These range from subclinical infections to febrile illnesses, including aseptic meningitis, and acute encephalitis. The prodromal phase commences with nonspecific influenza-like symptoms, including fever, headache, malaise, and vomiting, followed by an acute encephalitis phase and a spectrum of neurological manifestations. A notable proportion of cases present with polio-like acute flaccid paralysis [6]. Seizures and abnormal behaviors are prevalent in children [3,4,7,8,9,10], whereas febrile illnesses and meningitis are common in adults. The most frequently reported complications associated with poor prognosis are persistent seizures, motor neuron weakness, cerebellar signs, extrapyramidal disorders, arm flexion deformities, hyperextension of the lower extremities, cognitive impairment, language impairment, learning disabilities, and behavioral disturbances [11].

JEV is transmitted via the bite of an infected mosquito, mainly *Culex tritaeniorhynchus*, with an estimated 67,900 cases and 13,600 to 20,400 fatal cases annually [12]. Approximately 1% of individuals infected with the JEV develop acute encephalitis associated with high morbidity and mortality [13]. The incidence of this condition is relatively low, but when it does occur, the case fatality ratio is high, with reports of up to 30% mortality [14]. Viral virulence is not determined by genotype [15]. Indeed, it has been reported that virulence varies even among the same genotypes [15,16]. Furthermore, changes in the 3′-UTR have been reported, but these have not been reported to affect virulence [17]. They are also known to cause significant long-term complications in 30–50% of patients who recover from the disease, and no specific treatment has been identified thus far. Vaccines remain the most effective prevention method even today. In Japan, inactivated vaccines using cell-culture-derived viruses have replaced mouse brain-derived inactivated vaccines introduced in 2009. In addition to inactivated vaccines, live-attenuated and live vaccines using chimeric viruses with a yellow fever virus backbone have also been used globally [18,19].

In recent years, genotype 5 (G5) JEV has been detected in mosquitoes from South Korea [20,21] and China [22]. Consequently, JE cases have been reported in South Korea. G5 JEV was first identified in Malaysia in 1952. Current vaccines used globally are based on genotype 3 (G3), with ~90% homology at the amino acid level, whereas the vaccine efficacy against G5 might be reduced [23]. Although no G5 JEV cases have been detected in Japan, some studies have demonstrated that *Cx. tritaeniorhynchus* flies to Southeast Asia and Japan, indicating that JEV can spread to Southeast Asia [24,25]. In neighboring South Korea, reports of a genotype shift to G5 indicate that the distribution area may be expanding [26].

**Figure 1:**
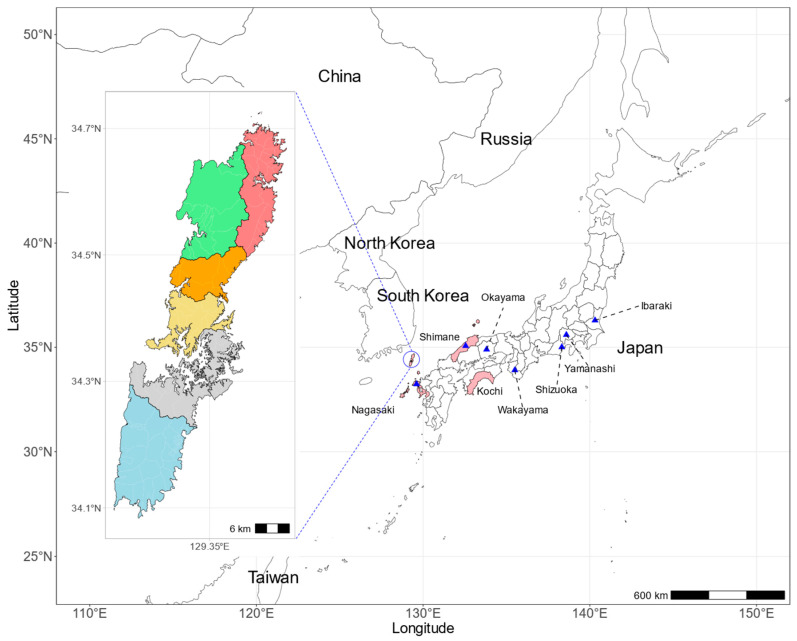
A map showing the location of Tsushima Island in Japan. Tsushima Island is located ~120 km from Kyushu Island. Tsushima has six administrative districts: Kamitsushima (red), Kamiagata (green), Mine (orange), Toyotama (yellow), Mitsushima (gray), and Izuhara (blue). Prefectures where swine sera were collected for virus isolation are indicated in pink. JE cases in 2016 were reported from the prefectures, of Ibaraki, Yamanashi, Shizuoka, Wakayama, Shimane, Okayama, and Nagasaki (blue triangles).

Before the introduction of the JEV vaccine in Japan in 1954, >1000 JE cases were reported annually. The prevalence of JE in Japan has dramatically declined since the introduction of the vaccine, and <10 JE cases were reported annually through 2016. Strikingly, 11 JE cases were recorded in 2016, 4 of which were diagnosed on Tsushima Island, a small island (population of 32,519 in December 2015) in Nagasaki Prefecture located halfway between Kyushu and the Korean Peninsula (Figure 1). The onset dates of the four cases were 19, 28, and 29 August and 15 September 2016, respectively. All four cases were presumed to have been infected on Tsushima Island and were active outdoors under normal circumstances, exhibiting independent survival. The occurrence of four JE cases in the same area within a single season was unusual. One patient died, two recovered, and one developed long-term neurological sequelae [27]. Japan has never experienced a JE outbreak on a remote island where pigs are not present, and there is a paucity of information on amplifying animals other than pigs. To ascertain the infection status and estimate the prevalence of infection in the post-outbreak period, a serosurvey was conducted on individuals residing on Tsushima Island. Furthermore, molecular epidemiology and seroepidemiology were employed to identify potential amplifying animals and the JEV that was prevalent during the outbreak.

## 2. Materials and Methods

### 2.1. Human Samples

From January to September 2017, blood samples were collected from 560 residents of Tsushima Island. The 560 residents represent 1.77% (95% CI, 0.69% to 2.85%) of the population on the island. All individuals participated in the study after face-to-face explanations, and their consent was obtained in paper form. Children were included if at least one of their parents provided consent on paper. The outdoor activity status and occupation were surveyed at the same time as blood collection using a questionnaire to determine whether there were opportunities for exposure to mosquitoes. Sera were separated within 24 h collection and frozen at −20 °C. Sera were diluted 1:10 in minimum essential medium (MEM; Sigma-Aldrich, St. Louis, MO, USA) containing 2% fetal bovine serum (FBS), heat-inactivated at 56 °C for 30 min, and diluted twofold from 1:10 to 1:640.

### 2.2. Plaque Reduction Neutralization Tests (PRNT)

Neutralizing antibodies against JEV were conducted using the PRNT method for JEV strains JaGAr01 (G3) and Muar (G5) [28]. Each JEV strain was grown as a Vero cell (JCRB9013; Japanese Collection of Research Bioresources Cell Bank). Human serum was diluted at twofold serial dilution and combined with strains JaGAr01 and Muar. The inoculated six-well plates were incubated at 37 °C for 4 to 5 days. The plates were fixed with ALTFix (Falma, Meguro, Japan), a formalin substitute, and stained with 0.1% methylene blue. The titers were defined as a 50% reduction relative to the nonserum control.

### 2.3. Wild Boar Samples

To evaluate the infection risk of JEV, sera from six wild boars were collected on Tsushima Island by the Nagasaki Livestock Hygiene Service in Tsushima and National Institute of Infectious Diseases between 13 June and 2 July 2022. Serum samples were separated within 4 h collection and frozen at −80 °C. Total RNA was extracted from wild boar sera using the MagDEA^®^ Dx SV RNA Kit (Precision System Science, Matsudo, Japan). cDNA was prepared using the PrimeScript™ II First-Strand cDNA Synthesis Kit (Takara Bio, Kusatsu, Japan) for reverse transcription polymerase chain reaction (RT-PCR) with oligonucleotide primers for detecting JEV RNA [28]. Real-time PCR was performed to detect JEV in the serum [29,30]. 

### 2.4. JEV Isolation and Strains

A molecular epidemiological survey was conducted to analyze circulating JEVs in the field. Swine sera were collected from Shimane, Nagasaki, and Kochi Prefectures between July and September in 2003 and 2008, 2015 and 2016, and 2018 and 2019, respectively. Blood samples from pigs were collected at slaughterhouses that were managed in accordance with legal regulations. For the collected sera, the samples positive for viral genomic RNA were selected by real-time PCR, and positive samples were inoculated into Vero cells for isolation. Sera were inoculated into the monolayer of Vero cells, kept in MEM containing 2.0% heat-inactivated FBS, and incubated at 37 °C for 7 days. The inoculated cells were blind-passaged at least twice. Infected cells and culture media were harvested when cytopathic effects (CPE) were observed and centrifuged at 3000× *g* for 10 min at 4 °C. The virus solution was stored at −80 °C until use.

JEV strains JaGAr01 (G3) and Muar (G5) were subjected to PRNT for seroepidemiological survey. Strain JA8015KC6 of the full-length coding region was sequenced using the primers listed in Supplemental Table S1. The amino acid identity of the predicted translated protein sequence of strains JA8015KC6 and JaGAr01 was 98.3% (Supplemental Table S2). Therefore, JEV strains JaGAr01 and Muar were used for neutralizing antibody tests. The complete nucleotides and amino acid similarity and identity are shown in Supplemental Table S2. Phylogenetic trees were generated using the Markov chain Monte Carlo (MCMC) method, MrBayes 3.1.2 [31], under the best-fit general time-reversible model of nucleotide evolution with gamma-distributed rate heterogeneity and invariable sites (GTR + I + Γ) using the jModelTest [32]. Two replicate Bayesian Metropolis–Hastings MCMC runs, each consisting of six chains of 10 million generations sampled every 100 generations with a burn-in of 25,000 (25%), resulted in 150,000 trees overall. Topologies evaluated by posterior node probabilities were based on 10 million generations for nucleotides and 1 million generations for amino acids, and estimated sample sizes over 100, implemented in MrBayes.

### 2.5. Statistical Analysis

Statistical analyses were performed with the stats (version 4.3.3) and rcompanion (version 2.4.36) packages in R version 4.3.3, and the ggplot and patchwork packages were used for data visualization. [33]. 

## 3. Results

### 3.1. Participants’ Profiles

A total of 560 individuals were included in this study. The age distribution was 1 to 98 years, and the median age was 63 years. A total of 351 samples were from females (62.7%). The island was geographically divided into six regions by administrative district, with 84 participants from Kamiagata, 156 from Kamitsushima, 26 from Mine, 28 from Toyotama, 90 from Mitsushima, 167 from Izuhara, and 9 unlabeled. Occupations were divided into seven groups: (1) healthcare workers, (2) desk workers and services, (3) security guards, janitors, and constructors, (4) agriculture, forestry, and fisheries, (5) homemakers and unemployed, (6) children, and (7) others (Table 1).

### 3.2. Seroprevalence of JEV Strains JaGar01 and Muar

**Figure 2:**
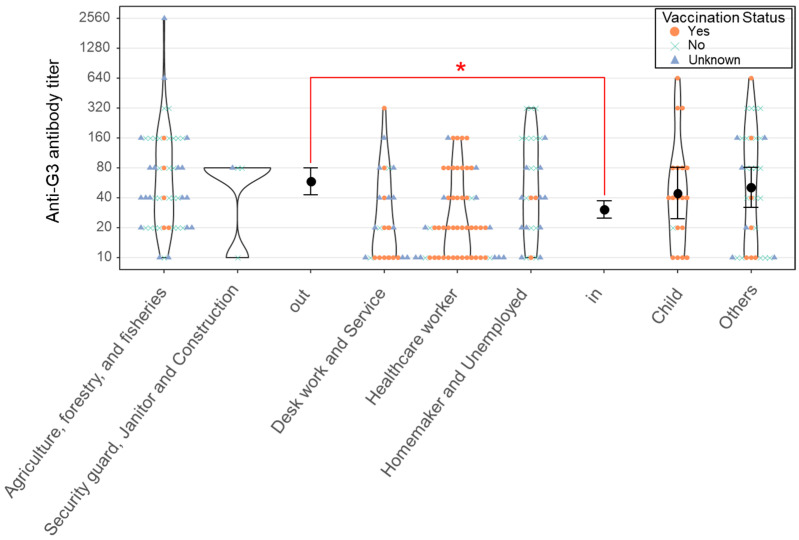
The violin and bee swarm plots in the graph show the distribution of antibody titers for each occupational category. The participants were divided into three groups based on their vaccination status: vaccinated individuals are represented by orange circles, unvaccinated individuals by aqua blue crosses, and those with unknown vaccination status by blue triangles. The geometric mean titers are represented by black dots, and their confidence intervals are indicated by the error bars around them. The occupational groups categorized under “out” include the two leftmost categories, representing outdoor work settings, whereas the “in” category includes the three central groups, representing indoor work environments. Welch’s *t*-test was used to compare the antibody levels between the indoor and outdoor groups, and *p* < 0.005 is indicated by a red asterisk.

The seroprevalence rates of neutralizing antibodies against JaGAr01 and Muar were 38.8% (217 of 560) and 24.6% (138 of 560), respectively. High anti-JEV neutralizing antibody titers of ≥320 were identified in 16 residents, including 3 children younger than 6 years with prior JEV vaccination, 2 adults in their 40s, and 11 adults older than 70 years (Figure 2). The antibody-positive elderly residents were uncertain about their JEV vaccination history, but it would have been >25 years ago even if they had been vaccinated. Furthermore, anti-JEV-specific IgM was not detected in any of the residents. All G5-positive sera were also positive for G3, and 136 of 138 sera positive for both genotypes had higher antibody titers against G3 than against G5. The remaining two serum samples had titers within the error margin at a twofold serial dilution.

To clarify differences in antibody titers according to the presence or absence of outdoor activities, antibody prevalence was compared by occupation. As a result, the groups of agriculture, forestry, and fisheries and security guards, janitors, and constructors, who are active outdoors, had higher neutralizing antibody titers than the groups of desk workers and services, healthcare workers, and homemakers and unemployed, who are often active indoors (Figure 2).

### 3.3. Field Investigation of Wild Boars

There were no livestock on the island, with only ~10 horses being raised. Therefore, a wild boar survey was conducted to identify amplified animals. As a result, no JEV was detected in the sera collected this time. In contrast, antibody titers against JEV were <10 to 640 times against strain JaGAr-01, <10 to 320 times against JA8015KC6, and <10 to 320 times against Muar strains. One of the three serum samples collected on 13 June 2022, was positive. Sera collected between 28 June and 2 July 2022, were positive for antibodies in all three wild boars (Table 2). Of the three strains, antibody titers against JaGAr01 were always the highest (Table 2).

### 3.4. Virus Isolation from Pig Sera and Phylogenetic Analysis

Sera collected from pigs in Shimane Prefecture in 2003 and 2008, from 140 and 90 samples, respectively, were inoculated into Vero cells for virus isolation. As a result, CPE was observed in 7 samples inoculated with 2003 samples and 1 sample inoculated with 2008 samples. In contrast, serum samples were obtained from 80 pigs in Kochi Prefecture in 2018 and 2019. A total of 10 PCR-positive pig serum samples (5 PCR-positive sera in 2018 and 5 in 2019) were used for virus isolation. Ten swine serum samples were inoculated into Vero cells, and CPE was observed in one sample each in 2018 and 2019. RNA was extracted from the culture supernatant, and PCR was performed with JEV-specific primers, amplifying a specific band. Amplified bands were sequenced and determined to be JEV. These isolates were designated strains JA5402Sh03SW101, JA5408Sh03SW107, JA5413Sh03SW112, JA5414Sh03SW113, JA5415Sh03SW114, JA5418Sh03SW117, JA5430Sh03SW129, JA5826Sh08SW55, JA8006KC44, and JA8015KC6 (Supplemental Table S2).

**Figure 3:**
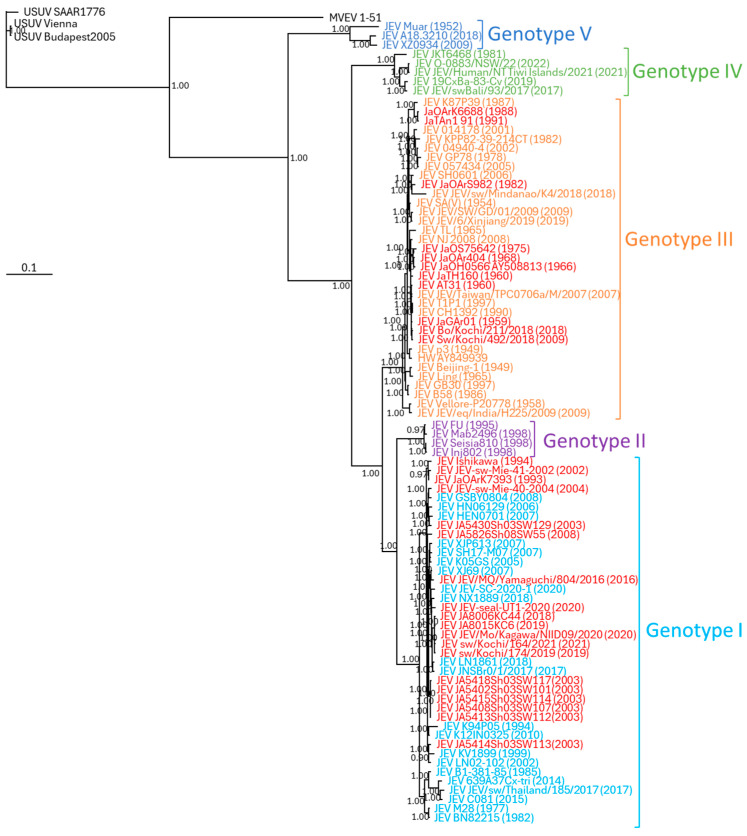
Phylogenetic tree, based on sequences of the entire coding region of JEV, generated by the Bayesian MCMC estimation method under the GTR + I + Γ model of evolution. USUV and MVEV are indicated by black characters. Genotypes I to V JEV are shown in light blue, purple, orange, light green, and blue, respectively. Red indicates Japanese isolations. Sample names are indicated by the virus abbreviation and strain name, and the isolation year is shown in parentheses. The sample details are listed in Supplemental Table S3.

Phylogenetic analysis was performed using the 10 virus strains obtained in this study and representative JEVs. The African Usutsu virus (USUV) and Australian Murray Valley encephalitis virus (MVEV) were utilized as outgroups in the phylogenetic analysis. The phylogenetic analysis demonstrated that JEV isolated from pigs in Kochi Prefecture in this study belonged to G1. Furthermore, these viruses exhibited a high degree of similarity to viruses isolated from pig sera in Kochi Prefecture in 2019 (strain JEV sw/Kochi/174/2019) and 2021 (strain JEV sw/Kochi/164/2021), pig sera in Kagawa Prefecture in 2020 (strain JEV JEV/Mo/Kagawa/NIID09/2020), and seal sera in Chiba Prefecture in 2020 (strain JEV JEV-seal-UT1-2020; Figure 3). In contrast, viruses isolated from pigs in Shimane Prefecture in 2003 and 2008 were divided into two groups, although the difference of amino acid between the two groups was minimal at 99.8%.

## 4. Discussion

This study revealed the seroprevalence of anti-JEV neutralizing antibodies after an outbreak of JE without a classic amplifying host. Tsushima Island residents who engaged in outdoor activities exhibited high anti-JEV neutralizing antibody titers, whereas low antibody titers were detected in desk workers, service personnel, and healthcare providers, with large clusters exhibiting a titer of 10. This phenomenon could be attributed to the lack of natural boosters because of low outdoor activity after JEV vaccination.

In this analysis, outdoor activities were speculated to be one of the causes of the increased risk of JEV infection. This was presumed to be an increased risk due to increased opportunities for vector mosquitoes to suck blood. *Cx. tritaeniorhynchus*, the primary mosquito vector of JEV has been confirmed on Tsushima Island [25]. Although there were no domestic pigs in Tsushima Island that amplified JEV, the presence of neutralizing antibodies against JEV in boars and the increasing serum titers over time suggested that wild boars serve as virus-amplifying hosts. A serosurvey conducted using wild boar sera, the blood correction timing of which is unclear, reported positive antibody titers against JEV in wild boars in the northern part of Okinawa Island [34]. This result also suggested that wild boars may function as maintenance or amplifying hosts for JEV.

In recent years, JEV, mainly G1, has been reported in Japan. In this study, viruses isolated from blood collected from pigs in Shimane Prefecture in 2003 and 2008 and from pigs in Kochi Prefecture in 2018 and 2019 were also G1 (Figure 3). Other JEVs isolated from pigs in Kagawa Prefecture in 2020, mosquitoes in Yamaguchi Prefecture in 2016, and a seal [35] at an aquarium in Chiba Prefecture were also G1. In contrast, the G3 virus was isolated from pigs and cattle in Kochi Prefecture, although very few [36]. Results of seroepidemiological surveys of Tsushima Island residents and wild boars suggested that G3 JEV might be circulating on Tsushima Island (Table 2). The serum antibody titers in wild boars did not rule out the possibility that G1 and G3 JEV were circulating in Tsushima Island and Kochi Prefecture [36]. It is necessary to keep a close watch on viruses circulating in wild boars on Tsushima Island.

**Figure 4:**
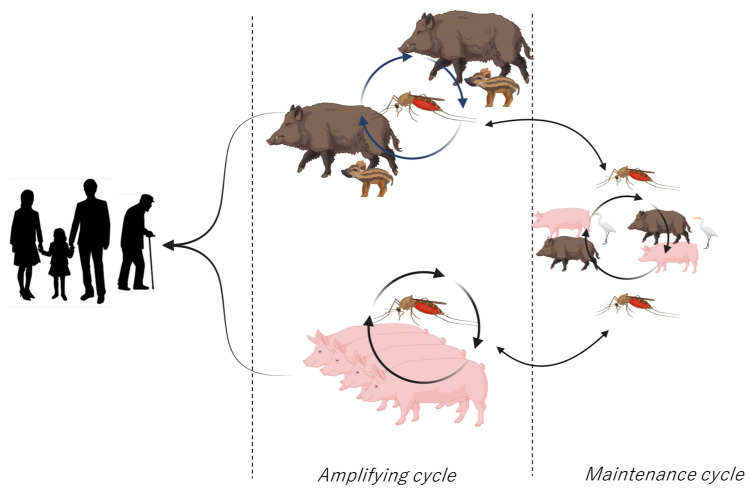
Schematic model presenting the amplifying cycles of JEV in the classic natural situation (**bottom**) [42,43] and on Tsushima Island (**top**). The content of wild boar piglets was imported from Freepik [44] and created using BioRender.com [45].

Tsushima Island is a quasi-national park and a known habitat for the endangered Tsushima leopard cat. Building construction and forest cutting are restricted in some areas of the park, and horses are the only livestock on the island. Approximately 90% of the area is forested, and no pig farms have been built on the island. Conversely, wild boars are widespread across the island. In 2016, >5000 wild boars were captured on Tsushima Island, and >8000 were captured in 2021 [37]. Wild boars are short-day seasonal reproductive animals [38,39,40] that annually supply susceptible animals to the environment. In most cases, births occur between April and July and serve as amplification animals in susceptible animals for JEV. In the 2022 outbreak in Australia, JE cases were reported in individuals around pig farms; hence, mosquitoes and pigs were deemed crucial in the outbreak [41]. Thus, these results suggested that JE outbreaks can occur in island environments featuring wild boars, even in the absence of pig farms (Figure 4).

A study on JE cases in Japan from 1982 to 2004 showed that only 20 of 361 (5%) cases had received the vaccine [46]. Fifty-one percent of patients were unvaccinated, and forty-one percent had an unknown vaccination history [46]. Although this study did not reveal G5 infection, it is assumed that G5 detection in South Korea and China will gradually increase the risk of G5 entry into Japan. Accordingly, JEV vaccination rates should be maintained at a high level. Because a long period had elapsed after vaccination, antibody titers were low in the elderly, even if they had received a routine JEV schedule. Nevertheless, the high JEV seroprevalence in the group with outdoor activity indicated high transmission rates, especially among the elderly, who are expected to be at high risk of JE and who should be administered booster doses.

## 5. Conclusions

This serosurvey of 560 residents on Tsushima Island revealed continued JEV transmission, even in the absence of pig farms. These findings indicated that wild boars could serve as amplifying hosts.

## Figures and Tables

**Table 1:** Age distribution and occupation of serum samples.

			Female	Male	Total
Age group		1–3	12	8	20
		4–6	8	9	17
		20–29	1	2	3
		30–39	8	1	9
		40–49	72	27	99
		50–59	70	33	103
		60–69	51	33	84
		70–79	64	51	115
		80–89	60	43	103
		90–98	5	2	7
		Total	351	209	560
Occupations	Healthcare workers	Doctors and nurses	106	8	114
		Medical staff	6	13	19
		Social workers	1	1	2
		Care workers	3	1	4
		Childminders	1		1
	Desk workers and services	Desk workers	24	40	64
		Services	6	7	13
	Security guards, janitors, and constructors	Security guards and janitors	2	2	4
		Civil engineers and constructors		4	4
	Agriculture, forestry, and fisheries	Agriculture	56	36	92
		Forestry		1	1
		Fisheries	4	7	11
		Agriculture and forestry		1	1
		Agriculture and fisheries	1	1	2
	Homemakers and unemployed	Homemakers	24		24
		Unemployed	33	36	69
	Children	1–3 years old	12	8	20
		4–6 years old	8	9	17
	Others	Self-employed	4	3	7
		Others	4	6	10
		Unknown	56	25	81
	Total		351	209	560

Homemakers: individuals responsible for the maintenance of familial life, including the performance of domestic tasks such as housework, childcare, and nursing care.

**Table 2:** Anti-JEV neutralizing antibody titers of wild boars in Tsushima Island.

					NT Titer		
Wild Boar No.	Sample No.	Sex	Correction Date	RT-PCR	JA8015KC6 (G1)	JaGAr01 (G3)	Muar (G5)
1	JA8475	Female	13 June 2022	Negative	<10	<10	<10
2	JA8476	Female	13 June 2022	Negative	<10	<10	<10
3	JA8477	Female	13 June 2022	Negative	80	160	160
4	JA8595	Female	28 June 2022	Negative	160	160	80
5	JA8596	Male	29 June 2022	Negative	320	160	160
6	JA8597	Female	2 July 2022	Negative	320	640	320

NT, neutralizing tests.

## Data Availability

GenBank accession numbers for phylogenetic analyses are available in Supplemental Table S3. Other presented data are available on request from the corresponding author.

## Supplementary Materials

The following supporting information can be downloaded at: https://www.mdpi.com/article/10.3390/v16081273/s1, Table S1. Oligonucleotide primers were used to amplify and sequence JEV isolated from swine serum. Table S2. Nucleotide homology and Amino acid identity of all viruses. Table S3. Sequences used for phylogenetic analysis.
